# Intracorporeal Cortical Telemetry as a Step to Automatic Closed-Loop EEG-Based CI Fitting: A Proof of Concept

**DOI:** 10.3390/audiolres11040062

**Published:** 2021-12-13

**Authors:** Andy J. Beynon, Bart M. Luijten, Emmanuel A. M. Mylanus

**Affiliations:** 1 Vestibular & Auditory Evoked Potential Lab, Department Oto-Rhino-Laryngology, Head & Neck Surgery, 6525 EX Nijmegen, The Netherlands; 2 Hearing & Implants, Department Oto-Rhino-Laryngology, Head & Neck Surgery, Donders Center Medical Neuroscience, 6525 EX Nijmegen, The Netherlands; bart.luijten@radboudumc.nl (B.M.L.); emmanuel.mylanus@radboudumc.nl (E.A.M.M.)

**Keywords:** cochlear implant, neural response telemetry, auditory cortical potentials, objective measurements, intracorporeal recordings, automated fitting, closed loop recordings, late latency responses

## Abstract

Electrically evoked auditory potentials have been used to predict auditory thresholds in patients with a cochlear implant (CI). However, with exception of electrically evoked compound action potentials (eCAP), conventional extracorporeal EEG recording devices are still needed. Until now, built-in (intracorporeal) back-telemetry options are limited to eCAPs. Intracorporeal recording of auditory responses beyond the cochlea is still lacking. This study describes the feasibility of obtaining longer latency cortical responses by concatenating interleaved short recording time windows used for eCAP recordings. Extracochlear reference electrodes were dedicated to record cortical responses, while intracochlear electrodes were used for stimulation, enabling intracorporeal telemetry (i.e., without an EEG device) to assess higher cortical processing in CI recipients. Simultaneous extra- and intra-corporeal recordings showed that it is feasible to obtain intracorporeal slow vertex potentials with a CI similar to those obtained by conventional extracorporeal EEG recordings. Our data demonstrate a proof of concept of closed-loop intracorporeal auditory cortical response telemetry (ICT) with a cochlear implant device. This research breaks new ground for next generation CI devices to assess higher cortical neural processing based on acute or continuous EEG telemetry to enable individualized automatic and/or adaptive CI fitting with only a CI.

## 1. Introduction

Since the clinical breakthrough of electrical auditory stimulation of the cochlea in the late 1970s, many studies investigating auditory-evoked auditory responses have been focused on its role as a tool to assess and/or predict auditory processing in cochlear implant (CI) candidates and recipients. In contrast to subjective behavioral assessment (e.g., tone- and speech audiometry), objective electro-physiologically obtained data are of importance for those subjects showing difficulties in obtaining reliable or consistent behavioral responses. Assessing the auditory function and determining electrical stimulation thresholds that are necessary to optimize individual CI processor fittings is not always possible in, e.g., very young pediatrics, mentally disabled adults, or, in general, difficult-to-test patients. Determining reliable subjective responses always requires, to a certain degree, some cooperation of the subject.

This prerequisite is one of the main incentives for many studies focusing on electrophysiological assessment to determine auditory neural activity in response to electro-stimulation in relation to individual behavioral responses. Many studies have investigated the predictive value of objective measures at different levels of the human auditory neural pathway, varying from peripheral electrically evoked compound action potentials [1,2,3,4], brainstem responses [1,5,6] up to middle- [7,8,9], late-latency obligatory cortical [10,11,12] and auditory change complexes [13,14,15,16] to obtain the pre-attentive mismatch negativity [17,18,19,20] to assess auditory discriminative skills. Besides exogenous auditory processing, endogenous higher-order cognitive P300 processing has also been reported in relation with CI performance [21,22,23,24,25,26]. Late-latency auditory processing might be a better indicator of the auditory perception, discrimination or even speech performance of CI recipients, since cortical potentials reflect auditory processing of the complete auditory neural pathway, combining bottom-up with top-down processing.

Electrophysiological assessment usually requires expensive electroencephalographical (EEG) devices for stimulation, response recording, data averaging and off-line analyses. However, besides the fact that these EEG systems are rather expensive as tools to record neural brain activity and have been applied for almost a hundred years [27], it is still a straightforward and easy applicable method to assess cortical processing in CI recipients in clinical settings.

However, although EEG devices might be relatively easy to operate, they are also sensitive to non-auditory responses; recordings might suffer from electrical field interferences caused by the length of electrode wirings, impedance mismatch between the recording surface electrodes due to fluctuating skin impedances or poor electrode montages that cause unwanted electrical noise. Additionally, other sources, such as internal physiological noise (biological EEG noise, ECG pick up) or magnetically induced disturbances (power line noise, radio frequency noise) often obscure the auditory target response. Apart from these arbitrary randomized responses, other non-auditory responses that are exactly time-locked with the stimulus, such as, e.g., myogenic (EMG) or eye-movement (EOG) responses, might be wrongly interpreted as neural activity in response to the auditory event. A possible solution to obviate some of these unwanted responses would be to perform these measurements under general anesthesia. However, besides its invasiveness, it is also not an option when auditory cortical and/or cognitive responses are involved, since the acquisition of these late-latency EEG responses requires a certain level of exo- and/or endogenous attention [28], especially when one is interested in central auditory cortical processing. 

Another practical disadvantage of a remote EEG recording system is that subjects are always directly connected to an EEG device by electrode wirings, which makes such a setup less optimal for, e.g., electrophysiological testing in young children. Finally, the success of recording optimal auditory responses also depends on the expertise of the clinician, who is accountable for, e.g., the correct montage of recording cables (electrode and cable positioning; avoiding possible cable loops; specifically for CI subjects; correct placement of stimulating CI-cable far away from recording electrodes or cables) and adequate parameter settings of the EEG system to reduce any disturbance of electrical stimulation artifacts. 

**Figure 1:**
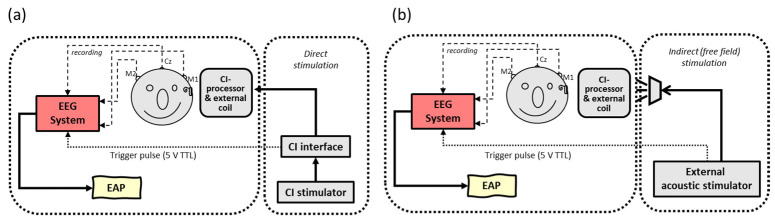
Schematic overview of conventional extracorporeal EEG setups with (**a**) direct stimulation: electrical pulses are generated by clinical CI software, directly streamed through the speech processor and presented to intracochlear electrodes vs. (**b**) indirect stimulation: sounds are presented to the CI speech processor via a sound field loudspeaker by an external stimulator. In both setups, an external EEG device is triggered by a stimulator to record time-locked electrically evoked auditory potentials (EAP).

In contrast to this ‘extracorporeal’ setup (i.e., the use of an external recording device outside the human body), the implementation of an ‘intracorporeal’ setup (i.e., the use of an internal implanted recording device) would be more convenient for patients as well as for clinicians in daily practice, leaving all previous mentioned disadvantages behind. Unfortunately, most electrophysiological measurements in CI are still performed with a remote evoked potential (EP) recording device, i.e., a clinical EEG system that is externally triggered by the CI system to obtain the responses. Stimuli are presented indirectly (via a loudspeaker) or directly (via the speech processor), as seen in Figure 1. 

Although all modern implant systems offer an external trigger signal for remote recording devices to capture electrically evoked auditory responses, it would be more convenient for the CI recipient and the CI clinician if all evoked potentials could be directly obtained from the implant itself, i.e., without an extracorporeal recording device.

Unfortunately, until recently, the only auditory evoked responses that could be recorded and averaged intracorporeally by a cochlear implant were electrically evoked compound action potentials (eCAP), cochlear microphonics and summating potentials using electrocochleography (ECochG). Since the introduction of eCAP telemetry in the late 90s, most CI manufacturers have developed specific hard- and software tools to evoke and record these eCAPs, better known as ‘neural response telemetry’ or NRT (Cochlear Corp., Sydney, Australia), ‘auditory nerve response Telemetry’ or ART (MedEl GmbH, Innsbruck, Austria) or ‘neural response imaging’ or NRI (Advanced Bionics LLC, Santa Clarita, CA, USA). 

In short, this telemetry functionality is based on the capacity of the implant to electrically stimulate the auditory nerve with an active (stimulating) pair of electrodes, while another (recording) pair of electrodes are used to capture and average the neural responses originated by the excitation of innervated spiral ganglion cells [29]. Since the biphasic stimuli of the cochlear implant also induce electrical artifacts within the recording time window, several paradigms based on forward masking have been applied to diminish these relatively huge artifacts from the real relatively small auditory neural response [30,31]. Nowadays, intracochlear telemetry has proven its practical value in clinical CI practice as a tool to define an optimal electrical stimulation profile to ‘map’ an individual electrode array. However, in contrast to what was initially expected, these eCAP data did not show an irrefutable one-to-one relation between the eCAP and behavioral thresholds, let alone its relation with speech perception [29,32]. The urge to investigate alternative objective measures other than those originating from merely the peripheral cochlear structures seems to become more relevant.

### Aim of the Study

Even though most contemporary cochlear implant systems feature a ‘closed-loop’ telemetry option for recording eCAPs, none of these systems can record long latency responses. One of the prerequisites to implement this is that the amplitude resolution of the internal CI amplifier should be sensitive enough to obtain responses in the domain of the cortical response. Since eCAPs have much smaller amplitudes than cortical responses, our hypothesis is that contemporary CIs should be capable of obtaining long latency EPs with these devices as well.

The main goal of this study was to examine the feasibility of using a CI device to capture and average electrically evoked auditory cortical responses (eACR) in CI recipients, by analogy with the recording of electrically evoked compound action potentials (eCAP). For the present proof of concept, a Nucleus Freedom 24RE cochlear implant (Cochlear, Sydney, Australia) was used for intracorporeal recordings, alluding to the idea that this proof of concept could be applied in other CI devices as well. Basically, two main prerequisites are of substantial importance to implement such an application with the present implants, i.e., firstly, the possibility to enlarge the recording time window beyond the first milliseconds of the eCAP and, secondly, sufficient implant amplifier sensitivity.

The NRT functionality of the Nucleus Freedom 24RE implant enables the capture of a maximum time recording window of 3.2 ms [29]. The recording resolution of the implant is dependent on the internal clock of the CI, the amplifier gain and implant full-scale amplifier voltage, with amplitude resolution depending on the number of observations, i.e., number of samples. According to the technical specifications, an amplifier gain of 70 dB would result in a voltage of 3.3 uV, as seen in Equation (1).


(1) $$\mathrm{Amplitude}\;\mathrm{resolution}=\frac{3.3\;\mathrm{uV}}{\sqrt{num\_trials}}$$

This implies that at least 250 averages are needed to obtain a resolution of about 0.2 uV for eCAPs. In contrast to (smaller) eCAPs, larger cortical responses require less amplitude resolution, so the CI amplifier seems—at least according to the technical specifications—to be able to adequately cover a sensitivity that is sufficient to obtain cortical responses with higher amplitudes with even less averages (e.g., *n* = 50). Given this, the main goal of the present study was the realization of intracorporeal cortical telemetry (ICT) to enlarge the temporal recording window of NRT to obtain larger higher-order cortical auditory responses.

## 2. Materials and Methods

The study comprised three consecutive experiments, i.e., two ‘in vitro’ experiments to verify the method of acquisition using artificial test signals and pre-recorded human EEG data, followed by an ‘in vivo’ experiment using real-time simultaneously acquired EEG from three CI subjects. 

### 2.1. Increasing the Time Window: Principle of Multiple Interleaved Concatenation of NRT Blocks

**Figure 2:**
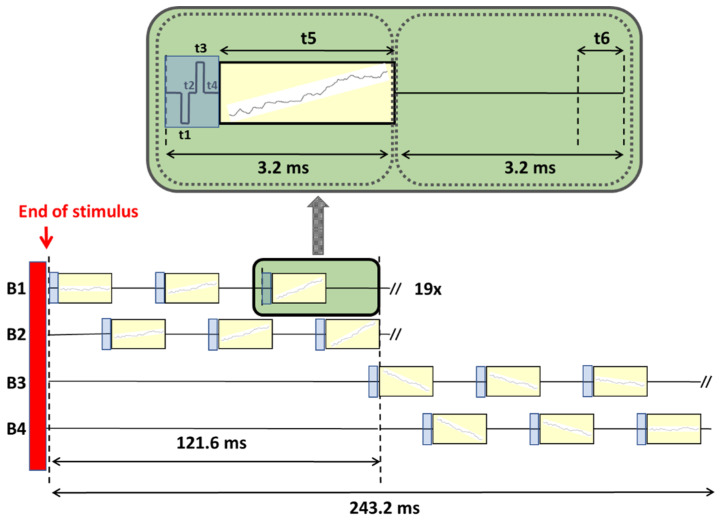
Visualized scheme of the recording principle to concatenate multiple standard ‘NRT blocks’ of 3.2 ms each, in 4 different memory buffers: Buffers 1 and 2 were interleaved in time, resulting in a recording time window of 121.6 ms. Another two extra buffers were added (Buffers 3 and 4) to obtain a window enlargement of 243.2 ms in total. Inset graph: biphasic pulse stimulus used in conventional NRT: t1/t3 = pulse width of 25 us; t2 = inter pulse gap of 7 us; t4 = recording delay of 90 us; t5 = 3053 us; t6 = 350 us (i.e., time required for data transfer). The vertical red bar before concatenation represents a 22 ms pre-recording time consisting of a pulse train stimulus.

To obtain electrically evoked Auditory Cortical Responses (eACRs), a total recording window of at least 200 ms post-stimulus is a crucial prerequisite. To accomplish this enlargement of the time domain, a custom built research application was developed (Labview, National Instruments, Austin, TX, USA) in combination with the Nucleus Implant Communicator research platform (NIC 2.0, Cochlear, Sydney, Australia) enabling the modification of default settings in the existing telemetry options. Typically, each NRT block (total length of 3.2 ms) consists of a stimulation time (i.e., t1–t3), followed by a recording delay (t4) and the actual recording time (t5) of the neural response, as seen in Figure 2. 

Instead of presenting default biphasic stimuli in time window t1–t3 (see Figure 2: in blue) after which the recording starts, a so-called ‘nil-stimulus’ was constructed (i.e., a stimulus without any charge or amplitude), followed by a recording with a sampling rate of 20 kHz (see Figure 2: recording windows in yellow). Since cortical potentials have an extremely slow amplitude slope within a 3.2 ms window, all recording windows were concatenated by connecting the average of a neural response at the end of t5 (last data point) to the start of t5 of the next block. Per NRT block, EEG data of each sampling window were captured and averaged per 10 EEG responses, manually exported and stored in a memory buffer (buffer B1), followed by the next 10 EEG responses that were captured, averaged, exported and stored in another memory buffer (B2), all in an interleaved way. By using ‘nil stimuli’, NRT blocks were only used for recording. Acquired data of two buffers was manually added up off-line, creating a total acquisition window length of 121.6 ms, each containing 60.8 ms (=19 × 3.2 ms) of EEG data. Outcomes from a previous pilot study, called ‘MPx’ (‘MPx’ has its origin in one of our initial experiments [33] using the monopolar electrodes MP1 or MP2 for recording (instead of stimulation) with the idea of implementing other alternative implanted ‘MPx’ recording electrodes), revealed that implant memory of this CI type was limited to a maximum number of 19 NRT data blocks, based on a fixed measurement delay of 90 us, 64 samples per ‘default NRT block’ and a 20 kHz sampling frequency). In order to capture a cortical P2 component as well, the same procedure was repeated for another two extra buffers (B3 and B4), increasing the recording window up to 243.2 ms post-stimulus. 

### 2.2. ‘In Vitro’ Recordings: Verifying the Interleaved Concatenation Paradigm (‘MPx’)

**Figure 3:**
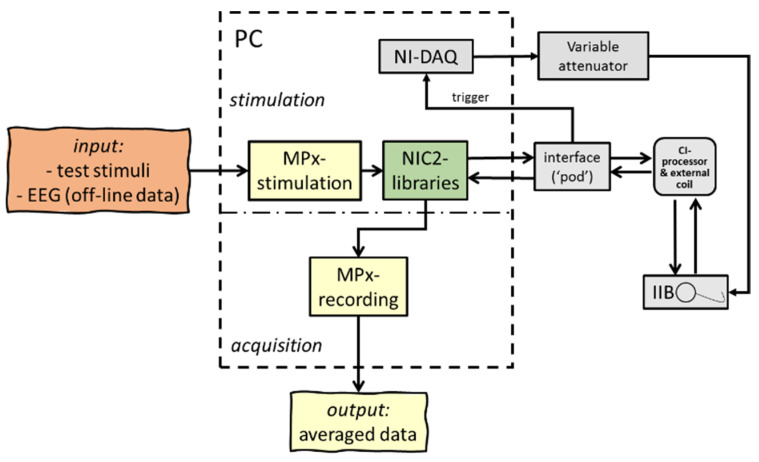
Schematic overview of hard- and software setup used in the ‘in vitro’ experiments; an extracorporeal cochlear implant emulator or ‘implant in a box’ (IIB) was used to simulate a patient. Artificial input data (test signals and EEG samples) were streamed through the IIB to confirm exact time-locked data processing.

To verify the principle of concatenation and merging of recorded data, an ‘in vitro’ setup was used with a CI emulator (or ‘implant in a box’, IIB) to simulate the patient, and the ‘MPx’ analyses were applied to process the acquired data. To test the ‘MPx’ telemetry program, a sinusoidal test signal was generated by a National Instruments Data Acquisition card (DAQ, type NI-PCI-6259, National Instruments, Austin, TX, USA) and served as a control stimulus to examine and confirm correct temporal synchronization (i.e., the length of an NRT window); additionally, pre-recorded human EEG was used as input. The recording was performed by applying so-called ‘nil-stimuli’; subsequently, the IIB captured NRT data blocks, according to the interleaved recording paradigm as described in Figure 2, and transmitted time-locked concatenated averaged output responses back via a speech processor to a graphical user interface. The data acquisition card was triggered by the POD to regulate synchronization between stimulation and recording. The amplified sinusoidal stimuli or artificial EEG signals were generated by the analog output of the DAQ, attenuated and streamed to the IIB for further ‘MPx’ back telemetry, as seen in Figure 3.

Besides using test signals, human EEG samples were also used as input for ‘MPx’ telemetry to verify the acquisition and averaging capacities of our telemetry modifications. Human EEG samples were previously obtained in normal hearing subjects with conventional EEG recording (see Figure 1) using a 5-channel EEG device (Jaeger Toennies, Germany). All cortical responses were acoustically evoked by averaging 2 × 50 short tone burst stimuli (1000 Hz, 22 ms duration, 2 ms rise-fall) that were acoustically presented in the sound field with a stimulation rate of 1.1 Hz and averaged with 0.1–30 Hz band pass filtering and an auto-reject level set at 50 uV, with a recording time window of 1000 ms, including a pre-stimulus time of 200 ms. Short bursts of 22 ms were chosen to avoid any overlap with our region of interest (ROI), i.e., latency area around the onset of the cortical N1P2 complex. All responses were recorded from Cz (non-inverting) referenced to mastoids M1/M2 (inverting) and a ground electrode placed at Fpz. Grand average responses were exported, saved as raw data and subsequently used as input for ‘MPx’ telemetry using the IIB for ‘in vitro’ verification.

### 2.3. ’In Vivo’ Recordings: Real-Time Stimulation and Simultaneous EEG Extra and Intracorporeal Recordings

**Figure 4:**
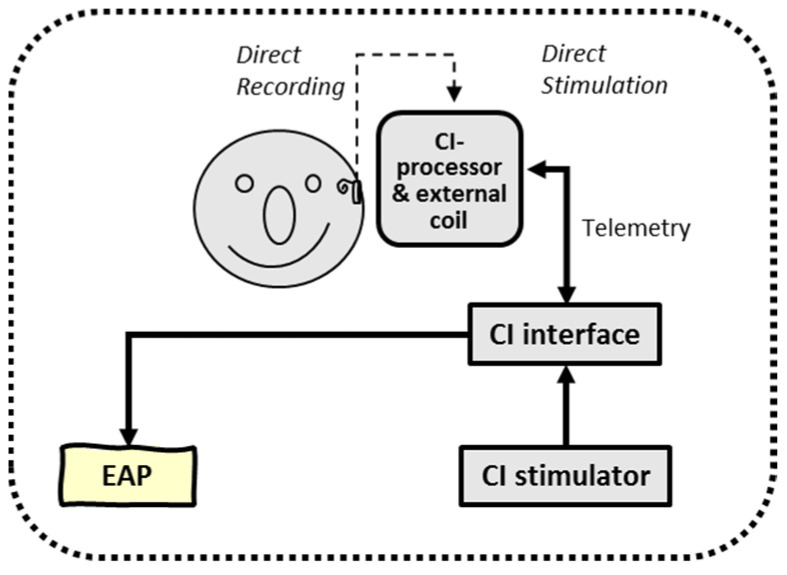
Schematic overview of intracorporeal telemetry; neural responses were obtained by direct intracochlear stimulation and recording (closed loop) using the back telemetry facilities of a cochlear implant.

The final experiment was the application of ‘intracorporeal cortical telemetry’ (ICT) in CI recipients without the necessity of an external EEG recording device (see Figure 4). Similar to NRT, using intracochlear electrode sites for monopolar stimulation and other neighboring electrodes to record neural activity, ICT typically uses intracochlear electrodes for (wide) bipolar stimulation and two extracochlear electrodes (MP1/MP2) for recording. In either case, NRT and ICT are typical examples of intracorporeal back telemetry: neural activity is obtained in a closed-loop CI system. 

Electrical stimulation was effectuated in a wide bipolar mode (BP+11), i.e., electrode 5 (basal) referenced to electrode 17 (apical) to approach a quasi-monopolar mode with a relatively broad electrical field to resemble conventional monopolar CI stimulation modes as much as possible. Like the acoustic tone burst stimulus, electrical biphasic pulse trains with the same relatively short duration (i.e., 22 ms) were chosen to avoid any interference of possible electrical stimulus artifacts with the obligatory N1P2-complex. All electrical pulse trains were presented with a stimulation rate of 900 pps (by analogy with default clinical CI stimulation rates), with 25 us phase width and 7 us inter-phase gaps at behaviorally determined comfortable loudness level (C-level) and presented with a rate of 1.1 Hz, similar to acoustic presentation rates. Subjects were three experienced post-lingually deaf adult CI recipients with complete insertions of the intracochlear electrode array (Nucleus Freedom 24RE CI device) without any intra- and postoperative complications. Electrode impedances of implanted and surface electrodes were checked and deemed to be within an acceptable range, i.e., <10 kOhm.

EEG recordings were executed under two simultaneous conditions, i.e., via conventional (extracorporeal) EEG recordings, and via intracorporeal cortical telemetry, both triggered by the same electrical biphasic stimulus trains. Recording parameter settings of the extracorporeal recordings were similar to conventional cortical measurements (see previous mentioned EEG settings in normal hearing subjects).

In contrast to conventional EEG recording using 2 or more surface electrodes on the scalp, the locations of the two extracochlear (‘monopolar reference’) electrodes of the CI device, i.e., MP1 (ball electrode, typically placed subcutaneously under the mastoid muscle) and MP2 (titanium implant housing) were now used for the disposable surface electrodes montage.

Electrically evoked ACRs were obtained with conventional EEG recording techniques (see ‘in vitro’ parameter settings), except that responses were derived from a recording configuration with the non-inverting electrode situated around the area of the implant housing (MP2) at the temporo-parietal area of the mastoid TP7/8, according to the 10–10 system [34]. The reference inverting electrode was located at C5/6, approximating the location of the subdermal monopolar ball electrode (MP1) of the CI.

**Figure 5:**
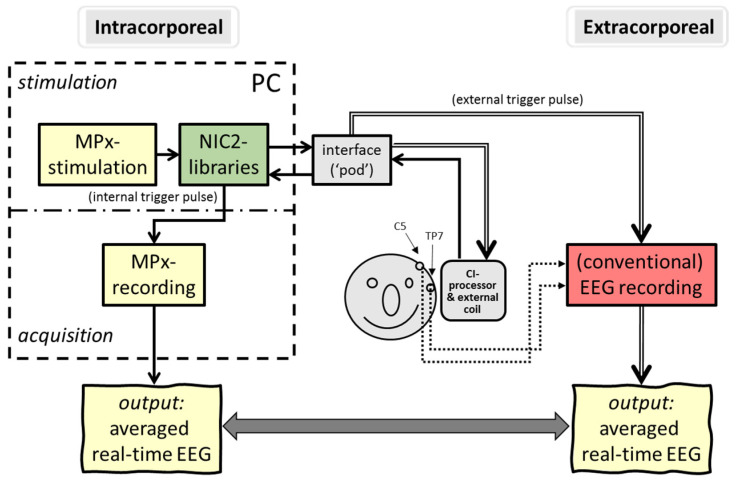
Simultaneous recording of eACRs; intracorporeal cortical telemetry (**left**) output was compared with conventional extracorporeal output (**right**).

Auditory evoked cortical responses were obtained and averaged by the implant, and data were processed through the ‘MPx’ software. Output was sent to a graphical user interface to show averaged electrically evoked auditory cortical response (eACR) in a window of 243.2 ms. For intracorporeal recordings, the two ‘monopolar’ electrodes MP1 and MP2 were used as recording electrode sites and thus inactivated for electrical stimulation. By choosing these electrode montages, comparison of data derived from the extracorporeal recordings and those derived from the subcutaneous (intracorporeal) MP1-MP2 recording sites was more realistic, as seen in Figure 5. Cortical N1-P2 detection components were visually interpreted by two independent experienced clinicians and analyzed for both recording conditions. Intracorporeal response was obtained, and morphologies were compared to simultaneously obtained extracorporeal responses.

## 3. Results

### 3.1. ‘In Vitro’ Recordings: Verifying the Interleaved Concatenation Paradigm (‘MPx’)

**Figure 6:**
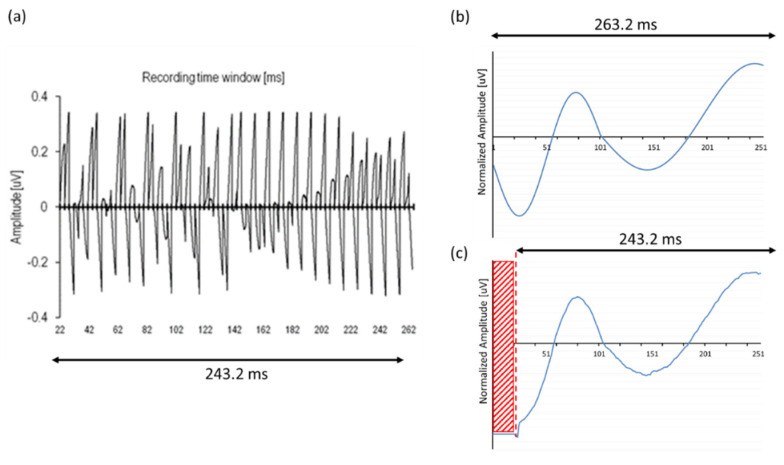
(**a**) Output of 72 zero-crossing in response to ‘nil-stimuli’ (i.e., stimulation without electrical charge) captured and averaged by four memory buffers, confirming an adequate concatenation of NRT recording blocks. (**b**) Artificial sinusoidal signals were used as test input to compare with (**c**) its output after ‘MPx’ processing. Note that output showed no recording in the first 20 ms (red area); X-axis corrected for latency shift.

The data show that simply concatenating consecutive ‘default NRT blocks’ of 3.2 ms each entailed the problem that the speech processor was not able to continuously capture and average data because of the lack of memory. Therefore, the enlargement of the total recording time window was split up into four memory buffers, assessing the correct timing of the concatenation. Stimuli without any charge (‘nil-stimuli’) were presented in an in vitro setup to record test signals and human EEG with an external CI (see Figure 3). To confirm the exact timing of the recording and, hence, the correct concatenation of multiple NRT blocks, the output of 76 consecutive ‘nil-stimuli’ confirmed a clear and perfectly time-locked response of 3.2 ms recording windows for each NRT block, i.e., 76 consecutive blocks of 3.2 ms consisting of noise peaks, as shown in Figure 6a. In addition, artificial sinusoidal signals were also generated by the DAQ, streamed to the IIB, recorded and processed with ‘MPx’ (see Figure 6b). Besides a slightly different offset, input–output curves showed similar response morphology, confirming adequate processing by the concatenation procedures, as seen Figure 6c.

**Figure 7:**
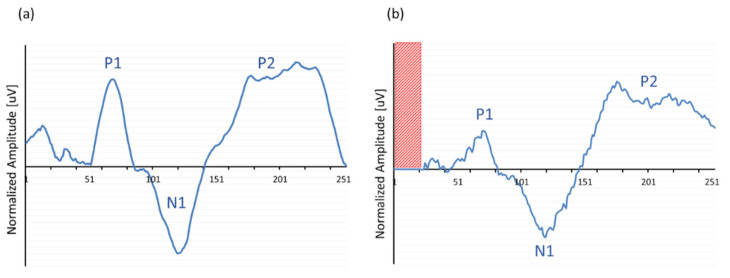
Typical example of an averaged acoustically evoked ACR in response to a 1 kHz tone bursts recorded with (**a**) a conventional EEG device and used as input for the (**b**) CI emulator (‘IIB’) to process ‘MPx’. Note that output showed no recording in the first 20 ms (red area).

A last verification of the ‘MPx’ concatenation process is shown in Figure 7. The hypothesis of this experiment was to show that the input of a more noisy ‘natural’ EEG signal would also result in a total concatenated response that would resemble the morphology of the original EEG signal in some way; therefore, previously acoustically obtained EEG data of a normal hearing subject in response to a 1 kHz tone was streamed as input for the extracorporeal IIB and subsequently processed and sent to the ‘MPx’ graphical interface. Amplitude was attenuated and normalized to adjust the absolute electric potential in the same range as those obtained in the conventional EEG recordings to compare the response morphologies. Although the output signal (Figure 7b) did not exactly mirror the original input (Figure 7a), it certainly approximated the typical slow vertex response morphology with its typical polarity change at latencies that corresponded to the original P1-N1-P2 components, confirming the ‘MPx’ programming of the window concatenation. 

### 3.2. ‘In Vivo’ Recordings: Simultaneous Extra- and Intracorporeal Recording

All recordings were repeated at least four times (4 × 50 responses), real-time averaged and off-line filtered between 1–20 Hz. All CI recipients showed the typical slow vertex cortical response morphology consisting of a negative trough around 80–120 ms (defined here as N1) and a positive peak around 160–220 ms (defined here as P2). Since the aim of the study was to develop a proof of concept, no statistical calculations were performed due to the low number of observations (only three CI recipients were involved). Table 1 shows peak latencies of the grand average of the slow vertex potentials N1 and P2 for each individual subject, as well as the peak-to-peak amplitudes of the N1P2 complex, obtained using conventional EEG equipment and intracorporeal cortical telemetry (ICT). 

**Figure 8:**
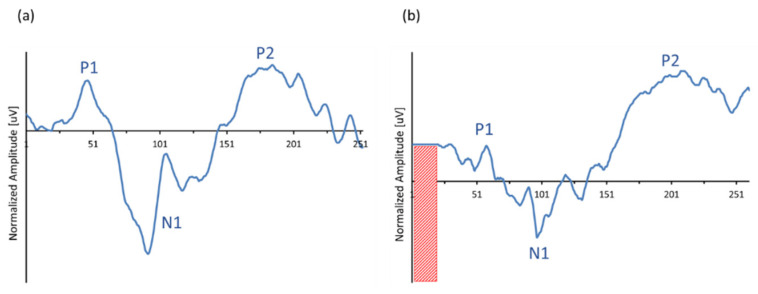
Example of typical morphologies of a simultaneously obtained extra- and intracorporeal recording in the same CI recipient (#2): (**a**) extracorporeal eACRs, obtained with a conventional EEG device, derived from surface electrode montage TP7-C5 vs. (**b**) intracorporeal ICT response, obtained by using the subcutaneously implanted MP1-MP2 reference electrodes as active recording sites.

Figure 8 shows the output recordings of the extracorporeal (Figure 8a) versus the intracorporeal recording (Figure 8b). In contrast to the recordings shown in Figure 7, Figure 8 depicts the grand average recordings of a CI recipient, typically showing simultaneous recorded eACRs obtained by a conventional EEG system vs. an ICT recording. Like in the ‘in vitro’ recordings, amplitudes were normalized and referred to the baseline starting point to compare the response morphologies of both recordings. Note that the ICT response was limited to a time window of 243.2 ms, inducing an artificial delay of 20 ms in latency for all peaks.

## 4. Discussion

Although most contemporary cochlear implant systems have the potential to perform intracorporeal recordings, telemetric functionalities are still limited to just measuring peripheral responses, i.e., eCAPs, cochlear microphonics and/or summating potentials. Late-latency potential recording still needs extra external EEG devices in combination with external stimulus generators (see an overview in Beynon [35]). A CI that can obtain, average and analyze late-latency electrically evoked auditory cortical responses would simplify clinical electrophysiological cortical testing and be useful for predicting behavioral thresholds or, more generally, evaluating auditory processing in CI recipients along the full auditory pathway.

The present study described a proof of concept to use a cochlear implant device as an EEG system; recording, capturing, averaging and analyzing EEG signals beyond the cochlea already seems to be feasible with present implant amplifiers. For the present CI system (i.e., Nucleus Freedom 24RE, Cochlear Corp., Sydney, Australia), a recording paradigm is proposed that is based on concatenating multiple NRT samples, resulting in longer recording window that comprise temporal domain of a cortical response. We have also shown that the amplifier range of this type of CI seems to be enough to average relatively larger response amplitudes of auditory cortical potentials. The latter makes recording relatively easy, compared to, for instance, brainstem responses with smaller amplitudes (<1 uV). After all, cortical potentials (1) have higher amplitudes and thus better signal–noise ratios (2) require less signal averaging and (3) show later response latencies, making them less susceptible to electrical stimulus artifacts coinciding with the ROI (i.e., response latencies of the cortical N1P2 complex) when relatively short stimuli (i.e., <80 ms duration) are used. Other studies have investigated similar setups to record early latency brainstem responses, implementing additional stimulus artifact-reduction paradigms that play a more substantial role in the early and fast-latency potentials [36,37]. However, when cortical responses are obtained with very short stimuli, such as those we applied (20 ms), the exponential decay of the stimulus artifact, in the order of tens of milliseconds, is fully extinguished around 80 ms (N1 latency).

Previously, intracorporeal experiments in animals have been reported by Heasman et al. [38]. They obtained auditory responses below 100 ms using monopolar recording electrodes in guinea pigs. Although their data suggest that ‘higher-order’ cortical recordings were also captured within 100 ms, their responses show atypical morphologies that—besides the different latencies—also did not seem to match the typical cortical morphology for guinea pigs. One of the reasons could be that the use of extracorporeal transcutaneous needle electrodes placed at the nape of the neck and the vertex recording the responses in combination with an extracorporeal implant amplifier (thus both extracorporeal recording and stimulation) was more susceptible to external noise interferences.

### Limitations and Suggestions for Future Advances in CI

The main practical clinical limitation of the present setup is that these recordings demand extra hard- and software adjustments. Besides, they appear to be time-consuming, i.e., at least four times longer than conventional EEG, due to the processing time needed to transfer and save buffer data because of shortage of implant memory. Fortunately, for implementation in future devices memory limitations are not insurmountable. Our initial expectations that ICT responses would exactly mirror the extracorporeally obtained responses appeared to be too optimistic; a possible explanation for these differences might be that real-time grand averaged (*n* = 50) EEG was obtained without any concatenation. In contrast, for ICT we combined 200 EEG responses (i.e., 4 × 50, see Figure 1), inducing extra EEG bias in each NRT window. Only when the EEG was consistent and very stable over time would the morphology be more identical (as visible in continuous sinusoidal test stimuli, see Figure 6c). 

An alternative way to increase the recording time window (or reduce measurement time) is to reduce the sampling rate per recording block (we used default high-resolution of 32 samples [29]). To recognize the N1-P2 component, a temporal resolution of one sample per 3.2 ms (≈312 Hz) would suffice, since the frequency of the slow vertex potential is <10 Hz. Thus, the most optimal and best practical solution would be to increase the telemetry recording window, e.g., into 400 ms by reducing the sampling rate. 

**Figure 9:**
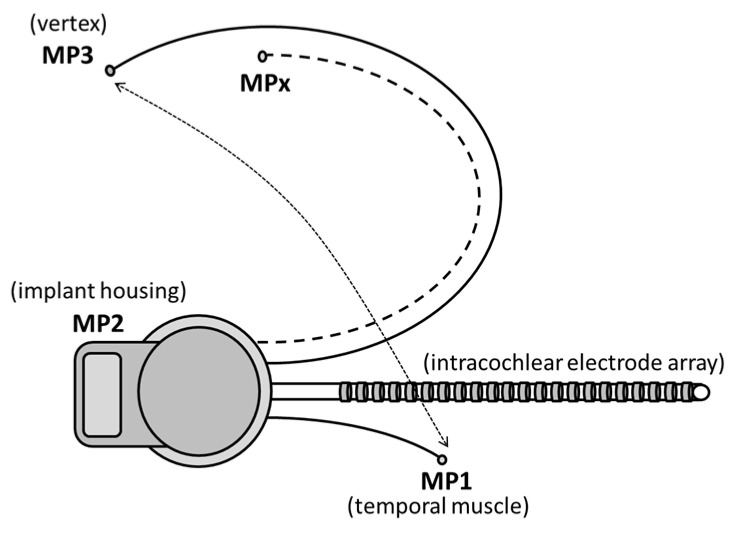
Schematic design of a CI with more than two subcutaneously implanted extracochlear electrode sites, e.g., MP3 at vertex (Cz), MPx on Fz, CPz, etc. to EEG (adapted from Beynon et al. [33]).

Another issue was the suboptimal recording sites of the present MP1/MP2 CI electrodes. After the first attempts to apply the ‘MPx’ concept in humans [33], we investigated the influence of sub-optimal recording sites and found that recording from scalp locations other than Cz mainly affected response amplitude, not latencies [39]. In the present study, we used the implant housing (MP2) referenced to the extracochlear ball electrode (MP1). The latter was intraoperatively placed below the temporal muscle as close as possible to the direction of the vertex, but it was limited by the length of the MP1 electrode, resulting in electrode placement around the C5/6 area. A possible solution for future systems to obtain better responses from specific scalp areas would be to implant one or more extra subcutaneous MP3, MP4 and ‘MPx’, electrodes, as seen Figure 9.

Somers et al. [37] recently described the use of an experimental percutaneous CI device that allows direct access to the implanted electrodes to perform closed-loop recordings; they performed ‘hybrid’ recordings to investigate optimal recording locations by connecting extracorporeal surface electrodes (Cz, Oz, P9/10) with intracochlear electrodes by making use of a temporary placement of a percutaneous connector. In contrast to our previous experiments to confirm adequate N1P2 recordings with surface electrodes at different scalp locations in response to bipolar electrical stimulation [39], the percutaneous CI setup of Somers et al. enabled them to combine scalp surface electrode recordings referred to different intracochlear electrode sites. Their results show better responses when these were derived from a scalp surface electrode at Cz referenced to one of the subcutaneous MP1 or MP2 electrodes. 

The idea to record EEG with a CI can be extended to other applications than the obligatory N1-P2-component; when subcutaneous electrodes are placed at Cz, Fz and CPz, other cortical potentials (i.e., auditory change complex, mismatch negativity and P300 potentials) could be acquired. The application of subcutaneously inserted scalp electrodes is not new; the placement of neurostimulators in epileptic patients in such a way that they electrically stimulate the brain at different cortical areas is well researched in the neuropsychiatry and neurophysiological fields [40,41]. The idea to apply more and longer electrode leads has already been implemented as well in CI recipients; the Neurelec Digisonic SP binaural CI device (Neurelec, France) makes use of one receiver on one side of the scalp and two stimulators (i.e., electrode arrays) implanted in both cochleae, demanding a subcutaneous placement of the electrode array across the whole scalp [42]. For the recording of EEG, intracranial electrodes are implanted in brain tissue to perform electrocorticography (ECoG) to monitor, e.g., ictal discharges [43] or to assess continuous audiovisual speech tracking in epileptic patients [44].

Besides a more ‘patient- and clinician-friendly’ setup, the implementation of intracorporeal cortical telemetry (ICT) in future CI devices would also incentivize clinicians to focus on auditory processing at higher order levels of the auditory system. It might boost cortical CI research in general and lead to the optimization and improvement of clinical patient care, similar to what occurred in the late 1990s with eCAP recordings. 

Remarkably, in the last two decades, there has been an increasing number of electrophysiological studies on the auditory processing of (sub)cortical neural processing. After all, higher order perception of speech is more related to (sub)cortical and cognitive linguistic processing than to peripheral intracochlear processing. To what extent cortical potentials might function to predict T- and/or C-levels in cochlear implant recipients is beyond the scope of this research, but a future with EEG-based automatic CI fitting seems within reach, even more so since peripheral auditory potentials have revealed meager outcomes with respect to electrical threshold prediction and speech perception until recently. In contrast, several cortical studies have reported the clinical value of threshold prediction in hearing-impaired and CI recipients [11,12,45]. 

The implementation of intracorporeal telemetry techniques will definitely pave the way for automatic CI fitting procedures steered by electrophysiological EEG data. The next challenge would be to combine recent developments in the neural envelope tracking of EEG and its relation with speech understanding [46,47,48] with research on the automatic recognition of cortical responses [25,49] for the next generation of cochlear implants. Besides the prediction of electrical thresholds or comfortable levels in the ‘difficult-to-test’ and pediatric population, new EEG research might enhance real-time neuro-feedback monitoring and/or automatic adaptive adjustments to optimize CI fitting schemes in the daily life of adult CI recipients, based on the advanced back-telemetry features of a CI.

## 5. Conclusions

The present study demonstrates a proof of concept that presents auditory evoked applications beyond the eCAP using the principle of the concatenation of multiple NRT windows with the aim to enlarge the recording time window for cortical response telemetry. With the present CI system (Cochlear Corp., Sydney, Australia) experiments confirmed the proof of principle, i.e., the feasibility of capturing and average intracorporeally auditory neural responses originating from higher cortical levels with a CI. For future applications, ‘patient- and clinician-friendly’ soft- and hardware modifications are necessary to facilitate intracorporeal recordings as well as the addition of one or more extracochlear electrodes to allow advanced EEG recordings of other cortical EPs with a CI. The present experiments hopefully stimulate and initiate new developments of CI devices, facilitating intracorporeal EP recordings and making automatic and real-time adaptive adjustments of individualized speech processor programming possible.

## Figures and Tables

**Table 1:** Peak characteristics of cortical N1P2 responses: extracorporeal (EEG) vs. intracorporeal (ICT) recordings (*n* = 3).

Subject	N1 Latency [ms]		P2 Latency [ms]		P-P Amplitude N1P2 [uV]	
	EEG	ICT	EEG	ICT	EEG	ICT
#1	92	80	217	189	10.9	9.8
#2	93	90	184	202	9.3	7.7
#3	81	110	189	194	11.5	7.9

## Data Availability

This research is a proof-of-concept study in a limited number of subjects, so no statistical analyses were performed. All in-vitro data (Figure 6) and individually obtained EEG data are reported in the article (Table 1). Raw EEG are available on request from the corresponding author.
